# Veterinary Titles

**Published:** 1895-07

**Authors:** 


### Veterinary Titles.

The near aspect of the coming meeting of the representatives of the veterinary colleges of North America lends interest and importance to the earnest letter of Professor Schwarzkopf on the all-important subject of a single title or degree for the American veterinarian. This has long been a vexatious question among American veterinary colleges, and new schools have only added more uncertainty and confusion as to the relative value of the different titles conferred by the various schools; and the disinterested public, on this technical point, have generously concluded that the veterinarians with a degree must be of a single kind and all well-equipped men, or of no worth at all, according to their individual experience with a single veterinarian whom they have employed upon a single occasion. When the degree of an American veterinarian represents a minimum course of instruction in well-equipped institutions, with trained instructors, of a period of study of three college terms of not less than six months each, then will the degree represent something tangible to the average American public.

